# Fluorescent probes as markers of cell envelope structure and function in halophilic archaea

**DOI:** 10.1038/s41598-026-61048-x

**Published:** 2026-07-11

**Authors:** Elisa Ravaro, David J. Burr, Xavier Marques, Andreas Elsaesser, Adrienne Kish

**Affiliations:** 1 Unité Molécules de Communication et Adaptation des Microorganismes (MCAM), Muséum National d’Histoire Naturelle (MNHN), CNRS, Paris, France; 2 Experimental Biophysics and Space Sciences, Institute of Experimental Physics, Freie Universität Berlin, Berlin, Germany; 3 Institute for Biology - Microbiology, Freie Universität Berlin, Berlin, Germany; 4 Institut UTINAM, UMR 6213 CNRS, Université Marie et Louis Pasteur, Besançon, France; 5 Unité Structure et Instabilité des Génomes (StrInG), Muséum National d’Histoire Naturelle (MNHN), Paris, France

**Keywords:** Biochemistry, Biological techniques, Biophysics, Cell biology, Microbiology

## Abstract

Prokaryotes, particularly those in extreme environments, are capable of diverse metabolic states resulting in altered cell envelope structure and function. However, these changes are difficult to assess as standard fluorescent probes are often incompatible with extreme conditions and/or extremophile cell physiology. Halophilic archaea present the challenge of near-saturated intra-/extra-cellular salts, high membrane potential, and extended survival in altered metabolic states including entrapment within salt crystal fluid inclusions. We evaluated the compatibility of six fluorescent markers of cell envelope stability and activity with two model species, *Halobacterium salinarum* and *Haloferax volcanii*. Redox activity markers alamarBlue and pure resazurin solutions, membrane potential probes MitoTracker Orange-CMTMRos and Rhodamine 123, and SYTO 9 and propidium iodide (LIVE/DEAD kit) to assess cell membrane integrity were evaluated for use in bulk (microplate reader) and cell-specific (microscopy) applications. Limitations of each probe were identified, clarifying the utilization of each based on cell physiology, growth phase, medium composition, and probe exposure time including extended timescales needed to simulate the environmental conditions of haloarchaea. Of particular note, propidium iodide behavior was unreliable leading to double-labeling of cells and false interpretation of cells as dead. These data provide important insights into the study of prokaryotes in non-standard conditions.

**Supplementary Information:**

The online version contains supplementary material available at 10.1038/s41598-026-61048-x.

## Introduction

The classical conception of microbial dormancy as a reversible state of reduced metabolic activity^1^ has been extended in recent years to include active but slow-growing microorganisms such as members of the ANME-2 group of anaerobic methanotrophic archaea from deep-sea sediments with doubling times measured not in hours but in months^2^. Identification of slow-growth metabolic states potentially extending microbial lifespans to thousands of years has even led to the proposal of ‘aeonophiles’ as a new category of extremophile^3,4^. This is particularly pertinent for microorganisms in extreme environments from halophiles in halite to permafrost bacteria to aerobic bacteria and archaea in deep-sea sediments for which prolonged survival durations up to millions of years have been suggested^5,6^. Measuring such states, however, requires metabolic markers that function both in extreme physicochemical conditions and with the unique cell adaptations of extremophiles. Reduced metabolic states of deep-sea sediment microbial communities have been analyzed using stable isotopes^7^, and bioorthogonal noncanonical amino acid tagging (BONCAT) has been applied during incubation of diverse extremophiles under in-situ conditions for deep-sea consortia^8^ as well as glacier communities^9^. For halophilic archaea, cell envelope markers need to function at salinities up to and including saturation. Also, they need to work with the accompanying cell adaptations, including the proteinaceous S-layer cell wall anchored into diether lipid membranes^10^, multiple genome copies per cell (polyploidy) in many species^11^, and ‘salt-in’ osmotic strategies.

Halophilic archaea inhabiting hypersaline environments face fluctuating nutrient and oxygen availabilities, changes in salinity and brine composition, and variable energy sources. Studies of model species such as *Halobacterium salinarum* (*Hbt. salinarum*) and *Haloferax volcanii* (*Hfx. volcanii*) show that acclimation to changing conditions relies on well-regulated stress responses and metabolic versatility (see reviews^12,13^ and references within). While these adaptive mechanisms are well-documented, the specific physiological and molecular underpinnings of reduced metabolic states, such as dormancy, remain poorly understood in haloarchaea. Metabolically inactive *Hfx volcanii* persister cells are genetically identical but phenotypically distinct, enabling survival under severe stress^14^. Entrapment of haloarchaea, commonly *Halobacterium*^15^, within halite fluid inclusions is an extreme stress condition that likely involves dormancy forms^16^. A state similar to Viable But Non Culturable (VBNC) may exist, which may also be distinct from persister cells^17^ or other novel reduced metabolic states yet to be identified. Haloarchaea have been suggested to survive entrapment, in some cases based on observations of cell morphologies extracted from ancient geological formations^5^. The reliance on lithological and mineralogical dating methods^15^, which do not directly date biological material, has led to controversies regarding the true age and viability of entrapped cells. In addition, observed morphologies may represent active cells, structurally intact but dead, metabolically inactive or dormant states. The physiochemical properties of the brine itself can enable preservation of dead cells^18^. The absence of validated in situ methods to study haloarchaeal cellular activity and structural stability has impeded testing these hypotheses.

The cell envelope, comprised of both the S-layer cell wall and lipid membrane, is central to distinguishing between microbial activity states. Especially, as its structural integrity and membrane potential are directly linked to metabolic functions, including redox gradients, and an usually high membrane potential in haloarchaea^19^. Novel dormancy states in low-nutrient, low-energy environments may involve cell envelope modifications^3^, such as recycling of membrane lipids rather than *de novo* biosynthesis, which has been observed for halophilic archaea and evolutionarily-related methanogenic archaea^7^ in energy-limited deep sediments. New tools are therefore needed to identify the metabolic state and structural stability of haloarchaeal cells, particularly in stress conditions.

Given the unique physiochemical properties of haloarchaeal cell envelopes, fluorescent probes targeting membrane potential, redox activity, or structural integrity are promising tools. However, few such probes have been rigorously tested or validated for use with haloarchaea, often only compatible under specific applications. Membrane potential detection using the cationic tetramethylrosamine-based probe MitoTracker Orange-CMTMRos (MitoTracker) was validated in *Hbt. salinarum* strain S9 for epifluorescence microscopy applications^20^. MitoTracker targets high membrane potential similar to that of mitochondria by binding covalently to protein SH-groups via its chloromethyl moiety, resulting in fluorescence quenching upon binding^21^. It is retained by cells even after reduction in membrane potential, enabling labeling over several generations as observed in *Hbt. salinarum*^20^. However, Maslov et al.^20^, did not evaluate MitoTracker-use with bulk fluorescence measurements such as platereader assays. Rhodamine 123, a lipophilic, cationic dye sensitive to both membrane potential changes and cellular ATP levels is an alternative probe for indicating metabolic activity or loss of metabolic function.

Redox activity is yet another method to differentiate active and inactive cells. Resazurin (blue color, absorbance maximum = 600 nm, low fluorescence) is reduced by NADH or other biologically abundant reductive species in presence of cytoplasmatic reductases^22^ to resorufin (pink color, absorbance maximum = 570 nm, high fluorescence) which is measured by either colorimetric or fluorometric methods^23^. Despite being widely used across bacterial and eukaryotic microorganisms, to date, the compatibility of resazurin (either as pure resazurin sodium salt or commercial alamarBlue solutions) with the chemistry of hypersaline conditions, its use over extended time periods, and its compatibility with reduced metabolic rates (likely for haloarchaea under stress conditions), have not been examined.

The few available fluorescent markers of cell membrane permeability are cytotoxic DNA intercalators including acridine orange, Hoechst 33342^22^ and the LIVE/DEAD kit, based on the membrane-permeable SYTO 9 and membrane-impermeable propidium iodide (PI)^24^. While the LIVE/DEAD kit has previously been used with haloarchaea^24^, studies reported discrepancies between colony-forming units (CFU) and PI-labeling^25^ coherent with findings from bacteria^26^. The underlying reasons for this discrepancy have not yet been identified. However, recent studies in bacteria have shown that PI staining often yields false-positives due to high membrane potential and extracellular DNA^27^, both of which are present in haloarchaea.

It is necessary to further test fluorometric methods, to ensure that different metabolic and structural states of halophilic archaea can be distinguished in the future. In particular, it is important to address exiting discrepancies and to identify cell envelope-specific fluorescent probes compatible with haloarchaea. As such, the compatibilities of six fluorescent probes were tested, including two membrane-potential indicators (MitoTracker and Rhodamine 123), two resazurin-based indicators for redox-activity (resazurin sodium salt and alamarBlue), and the LIVE/DEAD kit (SYTO 9 and PI). Compatibility was examined with *Hbt. salinarum* and *Hfx. volcanii*^10^, two well-studied model species since different haloarchaea physiologies, reflective of their respective environments, could result in differential cell envelope behaviors. *Hbt. salinarum* is a hypersaline specialist (optimal growth at 4.28 M NaCl) with rod-shaped cells under optimal growth conditions. In contrast, *Hfx. volcanii* is a moderate halophile with smaller, irregularly disc-shaped cells, tolerant to a wide range of salt concentrations (1.8–3.5 M NaCl)^28^ and higher concentrations of chaotropic Mg-salts.

The results of this study demonstrate both potential and limitations of using fluorescent probes to investigate cell integrity and activity in haloarchaea, using both bulk and cell-specific methodologies. The study parameters address the non-standard physicochemical and temporal conditions necessary to examine altered microbial cell states in extreme conditions. These findings have broader implications for understanding similar processes in both archaea and bacteria.

## Materials and methods

All experiments were performed as biological triplicates, with additional technical replicates as indicated.

### Culture conditions

*Haloferax volcanii* (DSM3757) was routinely grown under oxic conditions in HvYPC complex medium (see Table S1). For solid media 1.5% agar (w/v) was used. After autoclaving 3 mM CaCl_2_ was added.

*Halobacterium salinarum* strain NRC-1 was grown under oxic conditions in either complex media (CM) (see Table S1) or complex medium plus (CM+, derived from C. Evilia pers. comm) additionally containing 0.5% (v/v) glycerol, and 0.22 μm filter sterilized solutions of trace metals that were added after autoclaving. For solid media 2% agar (w/v) was added.

*Escherichia coli* strain BL21(DE3) was grown in Luria-Bertani media, with 1.5 % agar (w/v) added for solid media. Liquid cultures of all organisms were incubated at 37 °C, 180 rpm in glassware that had been rinsed in MilliQ water after washing and prior to sterilization to remove all traces of detergents. Cultures were grown to an optical density at 600 nm (OD600nm) of 0.5 (exponential growth phase), 1.0–1.5 (stationary growth phase; OD600nm of 1 is ~ 3.6 × 10^8^ cells/ml) or incubated for a further 2 weeks after reaching stationary growth phase (corresponding to decline phase).

For LIVE/DEAD experiments, attempts were made to produce cells with permeabilized membranes as a control for propidium iodide labeling, using a standard 60% ethanol treatment with both *Hfx. volcanii* and *Hbt. salinarum* following Leuko et al. 2004^24^. Both these strains of non-coccoid haloarchaea were not compatible with ethanol permeabilization resulting in cell lysis, coherent with the results from Leuko et al. 2004^24^. Survival was instead enumerated for live cells of control and treatment cultures by CFUs grown on nutrient agar to provide a basis for expected relative LIVE/DEAD labeling. Inoculated plates were dried in a laminar flow hood prior to incubation at 37 °C inverted inside sealed bags in the presence of a moistened paper towel forming a humidity chamber to avoid salt crystallization for 6–7 days. Viability was expressed as a percent survival compared to control cultures (N/N_0_ × 100). Statistical analysis of CFU counts was performed using the one-way ANOVA with pairwise comparisons using the Tukey post-hoc test, where *p* < 0.05 was considered significant.

## Fluorescent probe preparations and cell labeling

Stock solutions of fluorescent probes were prepared according to manufacturers’ protocols with either DMSO or MilliQ water as solvent (Table S2). Unless otherwise stated, all samples were incubated in the dark on a tube revolver/rotator (ThermoScientific reference 88881002, 20 rpm) according to the labeling protocol presented in Table S2. Where wash steps are indicated, the sample was centrifuged 3000 x g, 3 min, room temperature (RT), the supernatant discarded and the cell pellet resuspended in growth medium.

PI and SYTO 9 of the LIVE/DEAD BacLight Bacterial Viability Kit (Invitrogen reference L7012) were incubated according to Table S2 and Fig. S5.1 for imaging. Alternatively, for the ‘intra-storage’ labeling condition these fluorescent probes were kept (in the dark, RT, without agitation) in growth medium with haloarchaeal cells for 5 days and then imaged.

## Bulk measurements

All bulk measurements were performed using a POLARstar Omega (BMG Labtech) spectrophotometer running the Omega software (version 5.70 R2). Growth curves were measured in absorbance mode (Abs600nm), as was cross-reactivity of resazurin in deconstructed high-salt complex media for *Hfx. volcanii* and *Hbt. salinarum* (see Table S1). The shift in absorption was followed from the oxidized, blue resazurin (600 nm) to the reduced, pink resorufin (570 nm), compare Table S2 for exact wavelengths. Bulk measurements of relative fluorescence intensity (RFI) were performed in fluorescence mode.

## Microplate growth measurements

Growth curves were measured in transparent Nunc MicroWell 96-Well DeltaSurface plates (Thermo Scientific) with a sample volume of 200 µl as technical duplicates per biological replicate (*n* = 6). Cultures were diluted to OD_600_ = 0.05 with growth medium, mixed with the fluorophores at the indicated final concentrations (Table S2) and incubated over 70 h, 37 °C, 200 rpm, double-orbital shaking, with OD_600_ measured every 15 min for the first 4 h and then every hour. To prevent evaporation, empty wells were filled with water and the plates sealed with parafilm. Data of growth measurements was background corrected with the absorption of (organism-specific) complex growth medium at 600 nm. Pure DMSO (Invitrogen reference D12345) was incubated only with *Hbt. salinarum* at increasing concentrations (0.1, 0.2, 1, 2.5, 5 and 10% (v/v)) under the same conditions to test the growth impact of this commonly used solvent, since *Hbt. salinarum* is known to use DMSO as nutrient source under anaerobic conditions. In fluorophore-experiments DMSO was used in a final concentration of 0.1% (v/v). Growth measurement data with DMSO was background corrected by absorption of pure CM+. Statistical significance analysis of growth differences was performed using repeated measures ANOVA with pairwise comparisons with the Tukey post-hoc test, keeping the false positive risk at 5%. For this test *p* < 0.05 was considered significant.

## Fluorescence intensity bulk measurements

Bulk measurements of relative fluorescence intensity (RFI) were performed in black bottom Nunc MicroWell 96-well plates (Thermos Scientific, reference 137101) with a sample volume of 200 µl. Cultures in stationary growth phase were analyzed as technical duplicates of each biological triplicate (total *n* = 6) and mixed with the fluorescent probes in a final volume of 1 mL (Table S2). Samples were directly added to the wells and incubated for 4 h at 37 ˚C and 200 rpm in fluorescence mode. Measurements were performed with the appropriate fluorophore specific filter sets (Table S2). RFI of bulk measurements with cells were determined for each fluorescent probe by subtracting the raw value [measured as relative fluorescence units (RFU)] for cells in growth medium without the fluorescent probe from values of the equivalent sample with the fluorescent probe. For autofluorescence controls, RFU of pure growth medium was subtracted from RFU of growth medium with the fluorescent probe. This background correction allowed removal of any potential autofluorescence of the cells from the presented RFI data. In the case of cell-free medium controls, RFU of pure growth medium was subtracted from RFU of growth medium with the fluorescent probe.

### Resazurin reduction experiment

Cross-reactivity of resazurin was tested in deconstructed high-salt complex media for *Hfx. volcanii* and *Hbt. salinarum*. Solutions contained either only the salts needed for each organism (HfxS and HbtS) or the salts plus only one of the main complex organic nutrient sources (Table S1): Oxoid neutralized peptone (Pep N), Oxoid non-neutralized peptone (Pep NN) Bacto yeast extract (YE) and Bacto casamino acids (Cas). Test solutions were mixed with alamarBlue as indicated in Table S2 with (*n* = 6 technical replicates) and 200 µl of each sample were added into transparent Nunc MicroWell 96-Well DeltaSurface plates (Thermo Scientific). Absorption of the oxidized, blue resazurin, at 600 nm (Abs600nm) was measured using a POLARstar Omega spectrophotometer at 37 °C for 200 min and 200 rpm with meander corner well shaking and additional 300 rpm double orbital shaking every 15 min. In case of resazurin-reduction to resorufin, the absorbance shifts to 570 nm (pink resorufin). The data of resazurin cross-reactivity measurements was background corrected by the specific high salt solution without the fluorescent probe added, ensuring that the resulting dataset reflected only probe reduction.

## Cell-specific observations using epifluorescence/confocal microscopy

Epifluorescence microscopy was performed on cells immobilized on agarose gel slices using a Nikon TE300 inverted microscope, imaged with a 100x oil immersion objective (NA 1.3) in brightfield (phase contrast 3) and with fluorescence filter sets compatible with each fluorophore (Table S1). Images were acquired using a Photometrics CoolSNAP HQ camera and the MetaMorph software. Confocal Laser Scanning Microscopy (CLSM) of cells labelled using the LIVE/DEAD kit was carried out using an inverted LSM 880 Zeiss microscope equipped with a 40x oil immersion objective (NA 1.3) using the Zeiss Zen blue software. Images were analyzed using the FIJI software (Fiji is just ImageJ^29^. CLSM images were acquired as Z-stacks and therefore a Z-projection of median intensities for widefield images and of maximal intensity for fluorescence images was analyzed. Single epifluorescence-widefield images were analyzed, and background of all images was subtracted by a rolling ball radius of 20 pixels. Brightness and contrast were adjusted (for both CLSM and widefield images) in the same contrast range for all images of one fluorescence probe, to better show differences in fluorescence intensity of labeled cells. Labeled cells were counted across growth phases for MitoTracker and Rh123 and differences in labeling numbers were statistically analyzed with a one-way ANOVA test with pairwise comparisons by the Tukey Post-Hoc, where *p* < 0.05 was considered significant.

CLSM images of the LIVE/DEAD labeled cells were manually counted and labeling efficiency was calculated as total labeled cells (%) in proportion to the total number of visible cells in brightfield. Visible cells in areas of 200 × 200 pixels (20 × 20 μm) per image (separate for each image in brightfield, SYTO 9-filter, PI-filter) were counted manually. Statistical analysis of labeled cell counts was performed using the one-way ANOVA with pairwise comparisons using the Tukey post-hoc test, where *p* < 0.05 was considered significant. Fluorescence composites (PI- and SYTO 9 signal in one image) were used to identify number of double-labeled cells (yellow, orange and dark orange colors). Three different labeling protocols were used to simulate potential applications ranging from optimal to suboptimal environments for haloarchaea in stationary growth phase, such as entombment in halite fluid inclusions (Fig. S5.1). These included: (i) the standard labeling protocol as used for *E. coli* for stationary phase cultures (all cultures grown at 37 ˚C, 180 rpm), (ii) continuous exposure to SYTO 9 and PI throughout a secondary 5-day benchtop incubation (no shaking) after reaching stationary phase (intra-storage labeling), and (iii) labeling after the secondary 5-day incubation (post-storage labeling) compared to unlabeled storage control samples. While the latter two protocols followed the passage of cultures from stationary to decline growth phase, intra-storage labeling began in stationary phase whereas post-storage labeling only involved decline phase cells.

## Results

Three complementary approaches were used to assess the cell envelope structure and function of fluorescently labeled haloarchaea: [i] bulk measurements with UV/Vis spectrophotometry to evaluate fluorescence signal development, compatibility and potential cytotoxicity of the fluorescent probes and their solvents with archaea in hypersaline conditions; [ii] cell-specific fluorescence microscopy for information on labeling efficiency and specificity with respect to both species and growth phase; [iii] compatibility of the fluorophores with extended incubation times to account for long-term analyses with haloarchaea under sub-optimal conditions such as within halite fluid inclusions where cell activity and division rates would be reduced. Epifluorescence microscopy was used to determine the labeling efficiency (ratio of labeled to unlabeled cells) and labeling specificity of the fluorescent probes with cells of both haloarchaea. In addition, heterogeneities resulting from changes in cell physiology were evaluated using exponential, stationary and decline growth phase cultures (two weeks after entering stationary phase).

### Redox probes

**Fig. 1:**
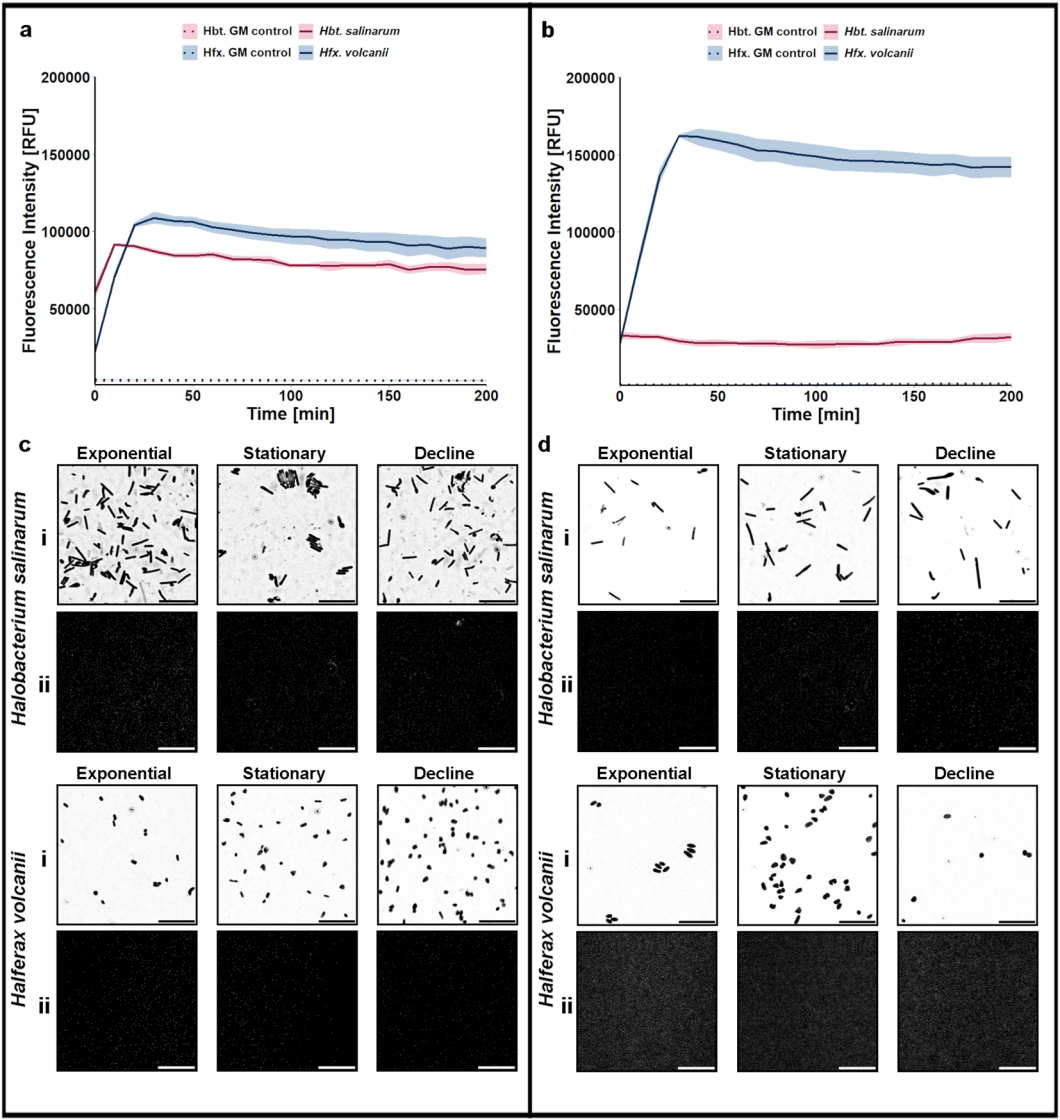
Compatibility of alamarBlue (**a**, **c**) and pure resazurin salt (**b**, **d**) with *Hbt. salinarum* and *Hfx. volcanii*. (**a**, **b**) Bulk measurements of relative fluorescence intensity (Ex: 544, Em: 590/10) with cultures of both organisms (stationary growth phase; solid lines) or solely in growth medium (GM = growth medium, corresponding to CM+ for *Hbt. salinarum* and HvYPC for *Hfx. volcanii*; dotted lines). Lines represent mean values, shaded regions represent the calculated standard deviation. Colors for alamarBlue samples (**a**, **c**) and pure resazurin samples (**b**, **d**) are identical since they represent the same detected molecule (fluorescent resorufin). Microscopy (**c**, **d**) of different growth phases per organism (exponential, stationary and decline phases) shown as widefield images (i) and fluorescence images (ii) that display the same image area, confirming that the fluorescent signals (white) are not cell-specific. Scale bars indicate 10 μm.

Compatibility of both resazurin formulations with different haloarchaea was evaluated using bulk measurements of RFI over time. Both alamarBlue and pure resazurin produced detectable fluorescent signals compared to growth medium blank controls (Fig. 1a, b). While the RFI of alamarBlue was similar for both haloarchaeal species tested, pure resazurin solution yielded 75% lower RFI in *Hbt. salinarum* compared to *Hfx. volcanii* (Fig. 1a) after signal stabilization roughly 20 min following probe addition. This suggests that alamarBlue additives compensate the variations in sensitivity of pure resazurin towards either the physiology of the haloarchaeal species or the composition of their respective growth media (salinity, salt compositions, nutrient and organics sources). AlamarBlue therefore appears to be more suitable to a range of haloarchaea. Observations with epifluorescence microscopy revealed that the fluorescent signals did not localize to cells (Fig. 1c, d). High background fluorescence intensity was observed for all samples, likely due to the production of reduced resorufin by cells, which was released into the extracellular medium, resulting in high background noise on fluorescence images (Fig. 1ci, di). This absence of cellular labeling is consistent with observations for mammalian cells, showing that resorufin does not accumulate intracellularly^30^ and is therefore not suitable for assessing cell-specific redox activity in haloarchaea by fluorescence microscopy.

Since no cellular labeling was observed and given that resazurin can be reduced by non-cellular reductants such as ascorbic acid or reduced glutathione present in cell-free growth media^31^, any artefactual effects with resazurin stemming from the growth media needed to be excluded. Reduction of alamarBlue was therefore measured at the maximal absorption wavelength for the oxidized molecule, resazurin (Abs600nm), after incubation in hypersaline solutions based on growth media of *Hbt. salinarum* and *Hfx. volcanii*. A decrease in absorption to 570 nm was expected if media components would reduce resazurin to resorufin. No reducing media components were identified for either media type (Fig. S1a), however different absorption intensities were observed for the solutions and alamarBlue (Fig. S1a). If non-neutralized peptone (PepNN) was used instead of recommended neutralized Peptone (PepN) the absorption of alamarBlue was lower, likely due to a more alkaline pH (Table S2) or specific peptone-components. To account for potential degradative processes occurring with standard benchtop storage of haloarchaeal growth media, the same sterile media were tested again after 3 mo of storage (Fig. S1b). All salt-based solutions with alamarBlue showed lower average absorption values after 3 months benchtop storage. These observations indicate that it is best to use freshly prepared growth media with any resazurin-based redox probes, especially if solutions are non-buffered or have more alkaline pH.

After reducing effects of the hypersaline growth media were excluded, the resazurin-based probes were tested for cytotoxicity in haloarchaea. AlamarBlue is marketed as a non-toxic probe, if used according to the manufacturer-recommended incubation times. However, incubation with resazurin over the course of microbial growth exceeds this time. Therefore, the potential toxicity of resazurin-based probes was tested with both haloarchaea over 60 h. Growth inhibition of *Hbt. salinarum* and *Hfx. volcanii* was observed with both probes (Fig. S2) and proven significantly different to the control cultures, with a two-way ANOVA and by using a Tukey post-hoc test (Table S3). This corresponds to findings^32^ that caution users of cell-type dependent levels of resazurin-tolerance. This underscores the need to limit incubation times in viability assays.

### Membrane potential probes

**Fig. 2:**
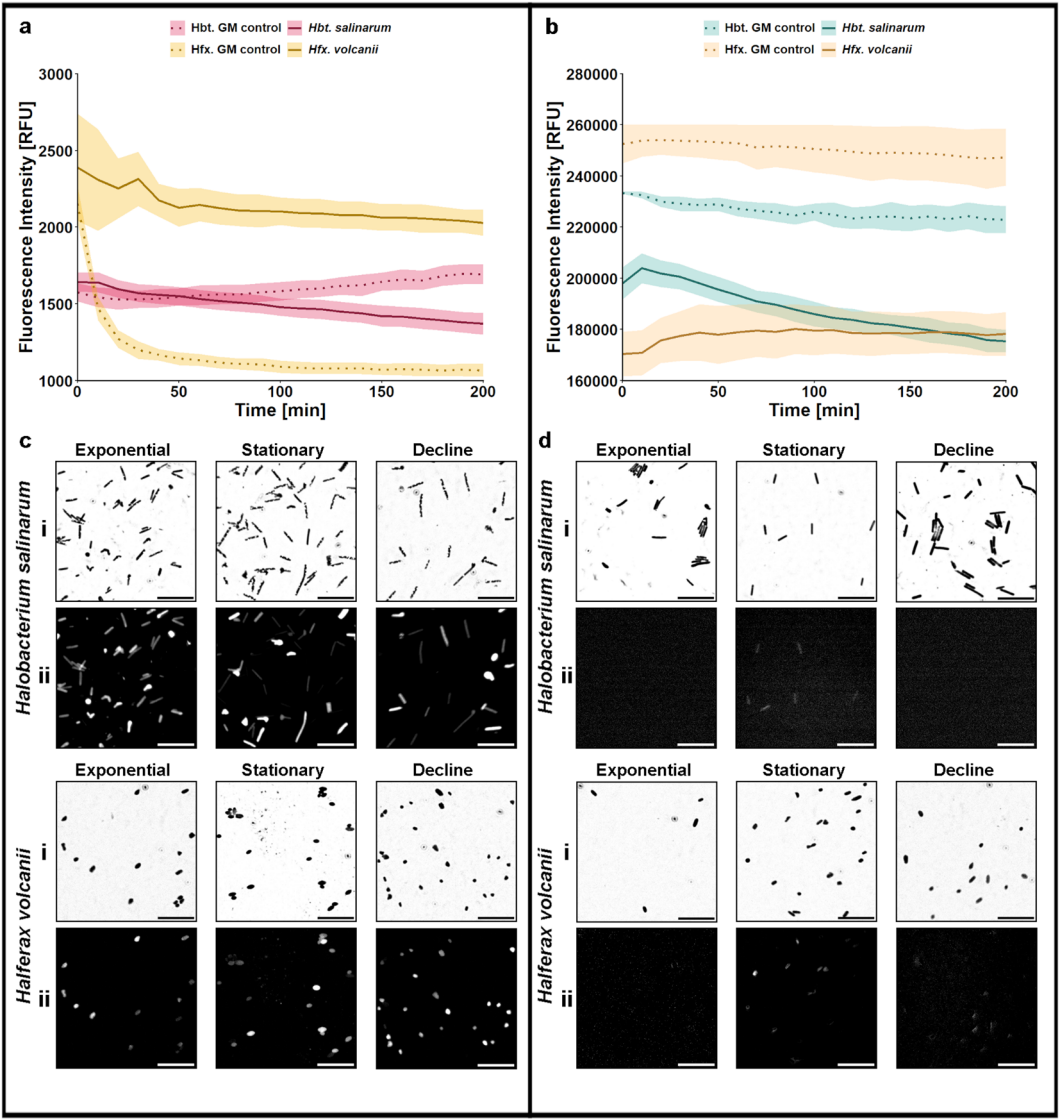
Compatibility of MitoTracker orange CMTMRos (**a**, **c**) and Rhodamine 123 (**b**, **d**) with *Hbt. salinarum* and *Hfx. volcanii*. (**a**, **b**) Bulk measurements of relative fluorescence intensity (Ex, Em wavelengths in Table S2) with cultures of both organisms (stationary growth phase; solid lines) or solely in growth medium (GM = growth medium, corresponding to CM+ for *Hbt. salinarum* and HvYPC for *Hfx. volcanii*; dotted lines). Lines represent mean values, shaded regions represent the calculated standard deviation. Microscopy (**c**, **d**) of different growth phases per organism (exponential, stationary and decline) are shown as widefield images (i) and fluorescence images (ii) that display the same area. Fluorescence signals in white show cell-specific labeling. Scale bars indicate 10 μm.

Bulk measurements were performed since literature currently does not describe measurement of RFI of the described membrane potential probes with haloarchaea. Measurements with both probes showed varied signal to noise properties relative to both probe type and haloarchaeal species. Stationary phase *Hfx. volcanii* and *Hbt. salinarum* cells incubated with MitoTracker produced relatively stable fluorescence signals over time (Fig. 2a). However, MitoTracker is an autofluorescent probe and therefore produced fluorescent signals in cell-free controls. Fluorescence in *Haloferax* growth medium (HvYPC) stabilized after 50 min whereas in *Halobacterium* growth medium (CM+) it increased over time. Additionally, cell-specific fluorescence intensities were 1.4-fold higher in labeled *Hfx. volcanii* compared to *Hbt. salinarum*. These observations complicate the interpretation of bulk measurements for *Hbt. salinarum*, where fluorescence was evaluated based on its dominant quenching effect and prolonged incubation times (> 2 h). It remains unresolved why the MitoTracker autofluorescence control showed this reduced RFI in *Hfx. volcanii* growth medium in comparison to the high RFI in the *Hbt. salinarum* growth medium. Under these conditions, MitoTracker RFI varied depending on both salinity and organism type.

Rh123 showed a stronger quenching effect than MitoTracker in haloarchaea, with cell-specific fluorescent signals being ~ 1.3-fold lower compared to Rh123 in cell-free controls (Fig. 2b). In the 1990s, similar quenching of Rh123 was reported in bacteria^33^, due to high probe concentrations, insufficient washing, high salinity, or particularly elevated K^+^ concentrations^34^. However, the precise mechanism of K^+^ interference with Rh123 has yet to be determined. The concentrations used here fall within the previously determined range for *Hfx. volcanii*^35^, following the current manufacturer-recommended wash steps. Notably, cell-free medium controls revealed higher Rh123-fluorescence in HvYPC (*Hfx. volcanii* medium) than in CM (*Hbt. salinarum* medium), coherent with the > 2-fold higher K^+^ concentration of HvYPC.

Epifluorescence microscopy was used to determine if growth phase specific differences in cellular labeling and therefore membrane-potential occur in either *Hbt. salinarum* or *Hfx. volcanii*. Non-uniform labeling of both species was observed for both membrane potential probes (Fig. 2c, d). These results were verified by enumeration, showing that the proportion of MitoTracker labeled cells was growth phase independent, remaining between 80 and 90%, with *Hfx. volcanii* showing slightly lower average numbers of MitoTracker-labeled cells than *Hbt. salinarum* (Fig. S3). However, differences in labeled cell numbers between growth phases were not significant for MitoTracker. Incomplete labeling with MitoTracker is consistent with the findings of Maslov et al.^20^ for *Halobacterium*, though direct comparisons are limited due to differences in microscopy techniques used between the two studies (confocal vs. widefield microscopy). The observed heterogeneity may reflect cell-to-cell variations in membrane potential kinetics (ion channel density, permeability, ATP levels), different metabolic activity within subpopulations or it might occur due to different probe accumulation inside the cells. Protocol optimization (for example, 1x washing instead of 3x before microscopy, incubation-times > 100 min) for enhanced signal discrimination is recommended. In contrast, Rh123 produced low fluorescence intensities (Fig. 2d), resulting in a labeling efficiency below 40% (Fig. S3). It is likely that either the strong probe quenching observed in bulk measurements (Fig. 2b) rendered the background fluorescence indistinguishable from labeled cells, or Rh123 might have been lost from haloarchaeal cells during the manufacturer-recommended wash steps intended to eliminate such background signal interference. While both MitoTracker and Rh123 are lipophilic cationic probes, differences in side groups result in MitoTracker being retained in cells, even after a loss of membrane potential, while Rh123 is lost. The non-permanent binding of Rh123 might also result in a loss of Rh123 probes during washing steps. Additionally, *Hfx. volcanii* cells export Rh123 via an ATP-driven transporter in the presence of an excess of amino acids^36^. Any effect on signal detection derived from either the probe concentrations or the use of generic filter sets simply reinforces the requirement of Rh123 for a high degree of preliminary optimization if it is to be used with haloarchaea.

Neither MitoTracker or Rh123 exhibited cytotoxicity in *Hbt. salinarum*, supporting findings of Maslov et al.^20^. for MitoTracker. The presence of MitoTracker conveyed a slight growth advantage during the late exponential growth phase, whereas Rh123 caused a slight decrease in the mid-exponential to early stationary growth of *Hbt. salinarum* (Fig. S2a). Statistical analysis of the growth curves showed that neither MitoTracker nor Rh123 impact growth of *Hbt. salinarum* significantly (Table S3). The slight growth advantage conferred by MitoTracker in the late exponential phase is unexpected and may reflect stabilization of membrane potential or metabolic shifts induced by the probe. Furthermore, it was shown by statistical analysis with the Tukey post-hoc test that the growth of *Hfx. volcanii* with MitoTracker is significantly slower (*p* = 0.0006) from its probe-free control, while Rh123 has no growth impact (Table S3). It remains uncertain why MitoTracker affected only one of the organisms. Further experiments are needed to elucidate the underlying mechanism.

The DMSO solvent used for both probes resulted in a concentration-dependent growth inhibition with statistical significance of *Hbt. salinarum* at concentrations over 2.5% (Fig. S4, Table S4) with total inhibition occurring at 10%, consistent with previous findings for bacteria^37^. This highlights the importance of stock solutions concentration (Table S2) to prevent DMSO cytotoxicity after addition to growth media. Concentrations of < 1%, which reflects the final concentration used in fluorescent probe experiments, have a cell-preserving effect preventing passage from stationary to decline phase. As *Hbt. salinarum* can use low concentrations of DMSO for anaerobic growth^38^, it is possible that cells underwent a metabolic switch that resulted in the observed prolonged survival in a reduced metabolic state. Therefore, attention should be paid to the DMSO concentration used with metabolic markers.

### LIVE/DEAD Viability Kit

**Fig. 3:**
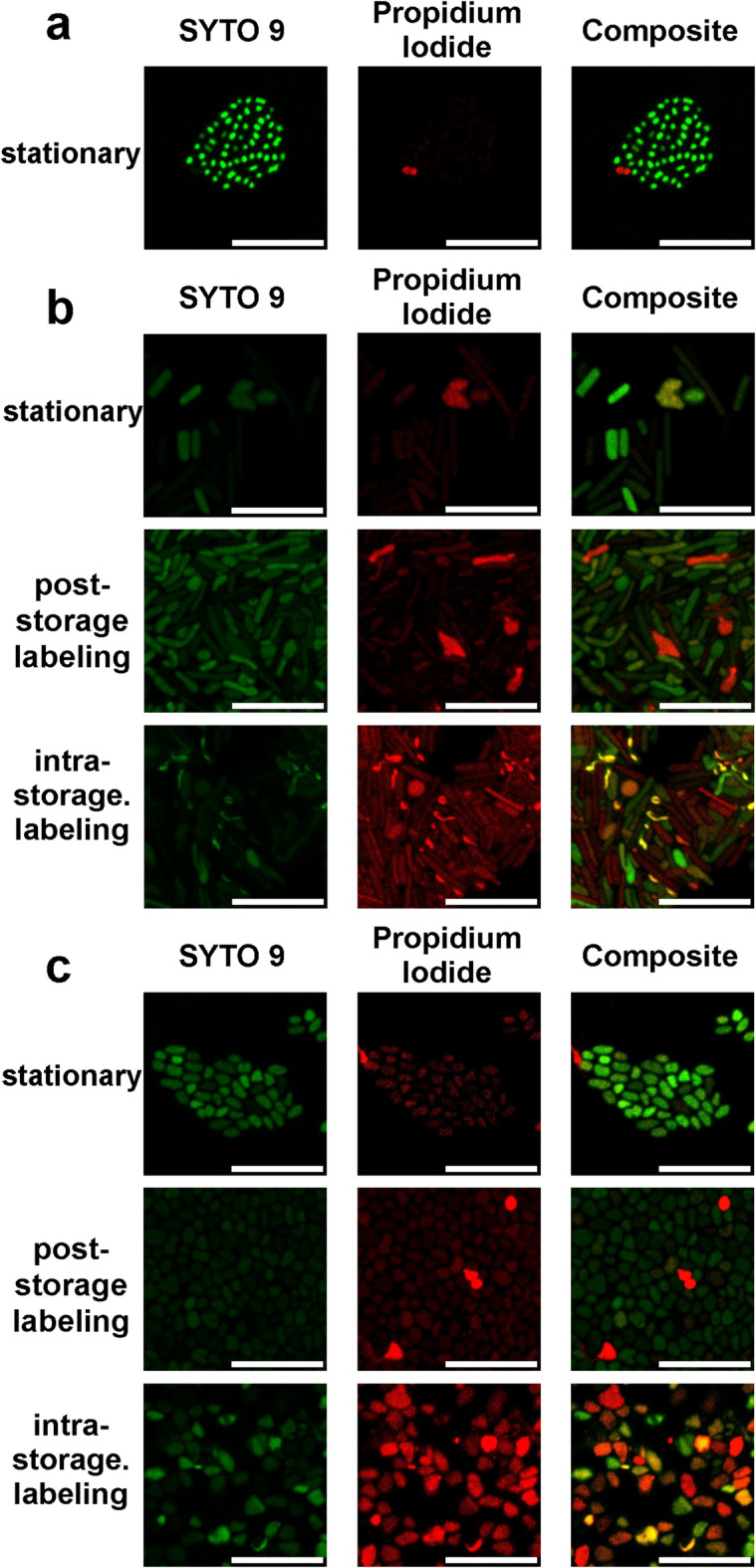
CLSM of various organisms incubated with the LIVE/DEAD BacLight Viability Kit (L/D). (**a**) *E. coli* labelled in stationary growth phase with L/D. (**b**) *Hbt. salinarum* and (**c**) *Hfx. volcanii* in stationary growth phase, labeled after 5 days of incubation (**post-storage labeling**) or incubated for 5 days with L/D (**intra-storage labeling**). Scale bars indicate 10 μm.

Prior to testing the LIVE/DEAD kit compatibility with haloarchaea, *Escherichia coli* cultures in stationary growth phase were used as a reference to validate kit functionality and establish a baseline for comparison. CLSM confirmed uniform labeling of live *E. coli* cells with SYTO 9, and dead cells (permeable cell envelopes) with PI (Fig. 3a). Quantification of labeling efficiency and specificity (Table 1) showed only 2% of *E. coli* cells remained unlabeled and that PI efficiently replaced SYTO 9 in dead cells as expected. In comparison, *Hbt. salinarum*, and to a lesser degree *Hfx. volcanii*, showed higher variability (standard deviation) in labeling efficiency in stationary phase, where both live and dead cells are expected. This is reflective of the polyploidy state of each species and high mutation rates in haloarchaea^39–42^.

**Table 1:** Effect of incubation conditions with the LIVE/DEAD BacLight Viability Kit on cell labeling of *E. coli*, *Hbt. salinarum* and *Hfx. volcanii* (*n* = 3 for each, with a minimum of 100 cells counted per biological replicate). Stationary phase cultures were either labelled immediately while decline phase cultures were stored for 5 days on the benchtop with labeling either at the end of the storage period (post-storage) or throughout the entire 5 days (intra-storage) (see Fig. S5.1). Total, single- and double-labeled cells were counted. Percentages were calculated as the number of fluorescent cells divided by total cell counts in brightfield and are presented as averages across replicates. High standard deviations in particular for *Hbt. salinarum* may reflect the known genetic variability of halophilic archaea.

Species	Labeling protocol	total labelled cells [%]	single SYTO 9 labelled cells [%]	single PI labelled cells [%]	double labelled cells [%]
*E. coli*	Stationary	98 ± 0	95 ± 0	5 ± 0	0 ± 0
*Hbt. salinarum*	Stationary	87 ± 24	85 ± 24	5 ± 5	9 ± 19
	Post-storage	74 ± 26	64 ± 17	7 ± 7	29 ± 17
	Intra-storage	44 ± 26	28 ± 36	53 ± 37	19 ± 19
*Hfx. volcanii*	Stationary	95 ± 8	1 ± 2	2 ± 16	97 ± 16
	Post-storage	5 ± 15	83 ± 11	4 ± 2	13 ± 9
	Intra-storage	94 ± 7	1 ± 2	49 ± 16	5 ± 16

Importantly, haloarchaeal cells displayed double-labeling with overlapping SYTO 9 and PI signals producing yellow-orange cells in composite images (Fig. 3b, c brightfield composite panels). This contradicts the expected probe behavior for dead cells as PI should displace SYTO 9 due to its greater DNA binding affinity^26^. Additionally, any remaining SYTO 9 signal should be locally absorbed within a ~ 10 nm range by PI due to Förster resonance energy transfer (FRET)^43^, as FRET-efficiency is inversely proportional to distance between energy donor and acceptor. Given that PI is assumed to be membrane-impermeable, a double label signal localized to a cell would therefore require either a live cell with PI bound to extracellular DNA in close proximity to the cell surface, or PI entering live polyploid cells at a slower rate creating a mosaic effect. This identifies PI as the source of the inaccuracies for use of the LIVE/DEAD kit to determine survival in haloarchaeal cultures.

To determine the extent of this phenomenon labeling specificity was calculated as single-labeled cells in proportion to the total number of labeled cells. *E. coli* cells in stationary phase showed no double-labeling. One-way ANOVA with Tukey post-hoc analysis confirmed that double-labeling ratios differed between *E. coli* and all haloarchaeal samples (F(5.25) = 35.97, *p* < 0.0001), whereas the phenomenon was particularly prevalent in *Hfx. volcanii* with 97% of stationary-phase cells displaying both fluorescent signals (Table 1), annulling any interpretation of cell viability. The statistical analysis confirmed that this effect was significantly higher in *Hfx. volcanii* than in all other samples (all *p* ≤ 0.0001).

Growth phase had a species-specific impact on labeling efficiency. Stationary phase cultures were compared to ‘post-storage’ cultures, which were stored at RT for an additional 5 days prior to labeling. The data suggests that unidentified physiological changes of *Hfx. volcanii* cells during storage after stationary phase produced over 7-fold fewer labeled cells, but those labeled had a higher specificity for either SYTO 9 or PI evidenced by a 2-fold reduction in double-labeled cells (Table 1). Conversely, comparable numbers of *Hbt. salinarum* cells were labeled in both stationary and post-storage, but with a 3-fold increase in double labeling.

To further investigate the potential for labeling haloarchaeal cells in reduced metabolic states as within halite fluid inclusions or during routine benchtop storage of cultures, the labeling time was extended to the 5-day storage period (‘intra-storage’ labeling, Fig. S5.1). This is an atypical application of the LIVE/DEAD kit as both SYTO 9 and PI are DNA intercalating probes, assumed to result in death of haploid cells during cell division. However, previous studies have successfully applied this method to polyploid haloarchaea in halite^36^, which suggests that survival of such cells is either a temporary phenomenon if indeed entrapped cells no longer undergo cell division, or the product of labeling only a sub-population of genome copies per polyploid cell, or false-positive signals. This extended labeling time resulted in a high heterogeneity for *Hbt. salinarum*, with a nearly 2-fold reduction in total labeled cells (Table 1, intra- vs. post-storage), which was statistically significant compared to stationary phase labeling of *Hbt. salinarum* and *Hfx. volcanii* (*p* ≤ 0.031). A corresponding reduction of double labeling under these conditions was indicative of higher specificity, with no visible changes in cell morphology evident in composite images (Fig. 3b). In *Hfx. volcanii* intra-storage labeling resulted in a high labeling efficiency and lower numbers of double labeled cells compared to stationary growth phase labeling (Tukey pst-hoc: *p* = 0.029 vs. *Hbt. salinarum* intra storage, *p* = 0.0001 vs. both haloarchaea stationary growth phase labeling), indicating that prolonged incubation with the LIVE/DEAD kit might help to reduce unwanted double-labeling.

Validating the interpretation of PI-labeled cells as dead is needed as membrane permeability in haloarchaea may not necessarily represent cell death, contributing to double-labeling. Standard ethanol treatment of non-fixed cells in both strains used here resulted in cell lysis rather than membrane permeability (see Methods) and no method has been found yet to produce a dead-but-intact (non-permeabilized) haloarchaeal cell. An alternative is CFU counts. As the DNA-intercalating action of both PI and SYTO 9 leads to inhibited cell division, CFU represent cells surviving LIVE/DEAD labeling (at least one genome copy remaining unblocked by DNA intercalators) or the respective storage condition. This method showed that labeling of stationary haloarchaeal cells did not result in lower CFU compared to unlabeled cells in the same growth phase (N/N_0_). Meanwhile was *E. coli* significantly susceptible (*p* = 0.025 vs. unlabeled control) to the toxicity of LIVE/DEAD (Fig. S5), likely due to a lower number of genome copies compared to the tested polyploid haloarchaea. Post-storage and intra-storage labeling however did result in a 40% reduction of *Hbt. salinarum* CFU in comparison to an unlabeled storage control (Fig. S5, storage control), indicating a physiological change over that storage period, which made *Hbt. salinarum* more susceptible towards the toxicity of DNA-intercalating probes, possibly allowed by its decreasing polyploidy^39–42^. However, an increase in CFU during the storage period of unlabeled *Hbt. salinarum* prevented any conclusions about actual dead-cell numbers in the samples analyzed by microscopy. In contrast a significant (*p* = 0.043) CFU reduction of 55% could be shown for *Hfx. volcanii* between the storage control and stationary samples (Fig. S5). Since CLSM dead cell counts (Table 1; single-labeled PI cells) indicated only 4% of post storage labeled *Hfx. volcanii* cells as dead, a difference of 50% in estimated numbers of dead cells emerges between these two methods. This discrepancy suggests that LIVE/DEAD staining underestimates dead-cell counts in *Hfx. volcanii.* In addition, this method seems to validate anecdotal evidence that *Hbt. salinarum* survives better and in fact continues dividing during benchtop storage compared to *Hfx. volcanii*.

## Discussion

Hypotheses of extended longevity and novel dormancy modalities in prokaryotes require new methods of investigation. This is of particular importance for extremophilic microorganisms. The effects of both the cell envelope adaptations of extremophiles and the physiochemical conditions required for their growth must be taken into consideration when measuring cell integrity and activity. Determining if extremophiles are true ‘aeonophiles’ relies both on analyses of natural samples and laboratory simulations of extreme conditions. New methodologies are required to monitor altered metabolic activities for haloarchaea entrapped within fluid inclusions of lab-grown halite and to determine if the cells are active, dormant, or dead. Fluorescent markers offer the potential for in-situ visualization of physiological changes. While this study focused on the compatibility of fluorescent markers for redox activity, membrane potential, and cell membrane integrity with haloarchaea, the results also revealed some principles widely applicable to other prokaryotes.

The feasibility of resazurin-based bulk redox activity measurements in halophilic archaea was validated, if both the chemistry of the fluorescent probe and the precise composition of the hypersaline growth medium are considered. However, it is of note that no fluorescence quenching of resazurin-based probes due to the high salt environment was observed in this study. Resazurin-based probes can be used for end-point assessment of redox-activity for example, after salt crystals with entrapped haloarchaea were rehydrated. If resazurin-based probes are chosen for bulk measurements, inclusion of extended controls (including growth medium, culture supernatant, and/or inactivated cells) is strongly recommended. It is to note that cell-free controls should always be used, to account for any cell-unspecific reduction of resazurin. Future studies could profit from reduction experiments with inactivated cells or growth media controls, where cells were removed after incubation and before any redox-indicator was added. Since fluorescence signals originate mainly from resorufin, which is known to accumulate extracellularly^30^, the probes are not recommended for cell-specific analyses like flow-cytometry or fluorescence microscopy. Since resazurin (alamarBlue) is known to be reduced by electron carriers such as NADPH/H^+^ that are essential to maintaining redox gradients for ATP production, a depletion of these carrier molecules might inhibit the cellular respiratory chain^32^. Long-term incubation is not recommended due to observed probe-toxicity.

For membrane potential, the results support the use of MitoTracker as a probe for bulk RFI measurements in haloarchaea, conditioned on preliminary compatibility testing with both the strain and suspension medium. Attention must be paid to its quenching upon binding to proteins, by selecting appropriate controls for interpreting fluorescence signals in bulk spectrometry. When the application protocol is optimized (1x washing instead of 3x before microscopy, incubation-times > 100 min) to prevent removal of weakly bound molecules, MitoTracker is the most versatile for use under a variety of conditions and different halophilic organisms. However, the exhibition of uneven fluorescence requires further investigation (like patch-clamp measurement of membrane potential and correlation of potential results to MitoTracker fluorescence intensity) to determine whether these differences reflect genuine variations in membrane potential or are influenced by other physiological factors. Furthermore, the overall weak fluorescence intensity of MitoTracker and its permanent binding to cellular structures^21^ prevents the monitoring of dynamic changes in membrane potential, highlighting the need for further research to identify more suitable probes for assessing membrane potential fluctuations. However, MitoTracker can still be used to track labeled haloarchaea, as Maslov *et* al. (2018)^20^ suggested in liquid cultures of *Hbt. salinarum* for extended incubation or alternatively in conditions such as inside salt crystals to observe degradation or conservation of cellular shapes. For other haloarchaea MitoTracker can be used in liquid cultures provided that no effects on growth speed are detected. While Rh123, the only tested probe sensitive to changes in membrane potential kinetics, has previously been used with *Hfx. volcanii* in flow-cytometry^36^, amino acid concentrations in the growth medium affect retention. The data here provide additional restrictions on Rh123 application emphasizing strict optimization of experimental conditions. The strong background fluorescence of Rhodamine 123 and its potential incompatibility with washing steps necessary to increase signal-to-noise ratios suggest that this membrane potential indicator should only be applied after species-specific protocol optimization, specifically regarding wash steps to remove unbound probes without leading to loss of cell-associated probes. Fluorescence bulk measurements showed that MitoTracker and Rh123 should be evaluated upon compatibility on a case-by-case basis, including both physicochemical conditions such as salinity as well as the microorganism itself.

Evaluation of the LIVE/DEAD kit to study cell membrane integrity over extended duration and in extreme conditions raised important concerns for its use, challenging the reliability of this type of labeling even under controlled laboratory conditions. High variability in labeling efficiency and specificity between the two tested haloarchaea species and *E. coli* highlights a strong species bias for the LIVE/DEAD kit within the prokaryotes, suggesting that haloarchaeal cell features such as the cell envelope structures (e.g., S-layer) or polyploidy may interfere with probe displacement or permeability. Even within haloarchaea, species bias was evidenced by differences between the two species in the labeling efficiency across various growth phases. The changes observed after culture storage also present a significant limitation for applications with haloarchaea as cultures are routinely stored in laboratories at room temperature after reaching stationary growth phase. In addition, the storage condition is also reflective of the hypothesized physiological state of *Halobacterium* cells entombed within the fluid inclusions of halite^16,44^. Taken together, these high variabilities rendered data interpretation with the LIVE/DEAD kit qualitative at best.

More importantly, PI failed to displace SYTO 9 consistently across different species, giving “false-dead” or double-labeled signals. The reliability of PI with some model organisms like *E. coli* resulted in its widespread application including with haloarchaea such as *Hbt. salinarum*^24,45,46^, but inconsistencies between viable cell enumeration and CFU plate counts^17^ have been previously noted under certain conditions. Contributing factors, such as the high-salt environment, the presence of extracellular DNA^27^, and particularly the elevated membrane potential in haloarchaea^26^, result in this false-positive PI labeling.

These results have implications far beyond halophiles by contributing to a growing body of literature for prokaryotes showing the limitations of PI for detection of dead cells. The observed differences in SYTO 9 and PI labeling are particularly restrictive for automated analyses such as flow-cytometry, independent of cell type. Furthermore, users should maintain set incubation times since extended exposure of cells to PI and SYTO 9 alters results, impacting reliability for batch analytics. Based on these findings, use of PI is not recommended as a dead-cell marker for mixed communities, such as environmental samples, or for single organisms that have not yet an optimized usage protocol (such as *Hfx. volcanii*). This applies to other extremophiles, as well as to mesophile species. The LIVE/DEAD kit has also used to identify VBNC cells in a wide range of prokaryotes including pathogens^47^, indicating a need for an alternative marker for this dormancy state. While the results presented here are tailored to halophiles, they demonstrate the absence of a direct ‘short-cut’ to identifying fluorescent markers to trace cell envelope integrity and metabolic states, particularly for extremophiles. Of particular interest is the compatibility of each fluorescent marker with the chemistries of hypersaline growth media components. The special experimental conditions like dormancy states of haloarchaea during entrapment require fluorescent markers to identify ‘ancient’ microbial life on Earth.

Taken together, this study provides new insights into evaluating cell envelope integrity and activity in extremophiles using both cell-specific and bulk methods. These findings open new doors to discover novel prokaryotic dormancy and cell preservation modes in extreme conditions.

## Supplementary Information

Below is the link to the electronic supplementary material.

Supplementary Material 1

## Data Availability

The datasets generated and analyzed for the current study are available from the corresponding author upon reasonable request.
