# Supplementary material for: Fluorescent probes as markers of cell envelope structure and function in halophilic archaea
Source: Sci Rep. 2026 Jul 11;16:21842. doi: 10.1038/s41598-026-61048-x (PMC13365526; doi:10.1038/s41598-026-61048-x)
Supplement: Supplementary file 1 — Supplementary Material 1 [file 41598_2026_61048_MOESM1_ESM.pdf]

Ravaro, E., Burr, D.J., Marques, X., Elsaesser, A., and Kish, A. Fluorescent probes as markers of cell envelope structure and function in halophilic archaea. Sci. Rep. (2026).

Supplementary Material

Table S1: Chemical composition and pH of high-salt solutions for *Halobacterium salinarum* and *Haloferax volcanii* based on their respective complex growth media CM/CM+ and HvYPC respectively. Solutions contained either only the salts needed for each organism (HbtS, HfxS) or the salts plus only one of the main complex organic nutrient sources: Oxoid neutralized peptone (Pep N), Oxoid non-neutralized peptone (Pep NN), Bacto casamino acids (Cas) or Bacto yeast extract (YE).

	HbtS	HbtS PepN	HbtS PepNN	CM	CM+	HfxS	HfxS PepN	HfxS PepNN	HfxS YE	HfxS Cas	HvYPC
pH	6.31	6.32	5.86	7.2	7.1	6.85	7.02	6.99	6.44	6.69	7.1
NaCl [M]	4.28	4.28	4.28	4.28	4.28	2.46	2.46	2.46	2.46	2.46	2.46
$MgSO_4 \cdot 7\ H_2O$ [mM]	81.2	81.2	81.2	81.2	81.2	85.2	85.2	85.2	85.2	85.2	85.2
KCl [mM]	26.8	26.8	26.8	26.8	26.8	56.3	56.3	56.3	56.3	56.3	56.3
$C_6H_5Na_3O_7 \cdot 2\ H_2O$ [mM]	10.2	10.2	10.2	10.2	10.2						
$MgCl_2 \cdot 6\ H_2O$ [mM]						88.5	88.5	88.5	88.5	88.5	88.5
Non-Neutralized Peptone (Oxoid REF LP0037)			1 % w/V					0.1 % w/V			
Neutralized Peptone (Oxoid REF LP0034)		1 % w/V		1 % w/V	1 % w/V		0.1 % w/V				0.1 % w/V
Bacto Yeast Extract (gibco, REF 12750)									0.5 % w/V		0.5 % w/V
Bacto Casamino acids (gibco, REF 223050)										0.1 % w/V	0.1 % w/V
Tris HCl (pH 7.5) [mM]											12
Glycerol					0.50%						
$ZnSO_4 \cdot 7\ H_2O$ [mM]	5.97	5.97	5.97		5.97						
$MnSO_4$ [µM]	21.9	21.9	21.9		21.9						
$CuSO_4 \cdot 7\ H_2O$ [µM]	2.73	2.73	2.73		2.73						
$FeSO_4$ [mM]	23.03	23.03	23.03		23.03						

Table S2: List of fluorescent probes used in this study, their biochemical function and utilization protocols, especially highlighting optimal excitation (Ex) and emission (Em) wavelengths as well as the wavelengths for Ex/Em used with each method (microscopy and platereader).

	Supplier and product reference	Chemical reaction	Targeted cell envelope function	Solvent type	Stock solution	Storage temperature	Working solution (Final concentration)	Preparation for microscopy	Optimal Ex/Em wavelengths [nm]	Ex/Em filter (POLARstar Omega spectro-photometer)
**Resazurin**	Invitrogen (ThermoFisher) Ref: R12204	Reduction from blue, non-fluorescent resazurin to pink, fluorescent resorufin [-O_2] *	Redox activity	water	20 mM	-20 ˚C	100 µM	Incubation with 8x10E7 cells for 240 min at 37 ˚C, then concentrated by centrifugation (3 min, 3000 xg, RT) to 8x10E8 cells<br><br>Ex/Em filter cube of Nikon Epifluorescence Microscope: Ex: 560/25 Em: 607/40	Ex: 570 Em: 580	Ex: 544 Em: 590/10
**alamarBlue**	Invitrogen (ThermoFisher) Ref: DAL1025	Reduction from blue, non-fluorescent resazurin to pink, fluorescent resorufin [-O_2] *	Redox activity	Ready to use solution	10x	4 ˚C	1x	Incubation with 8x10E7 cells for 240 min at 37 ˚C, then concentrated by centrifugation (3 min, 3000 xg, RT) to 8x10E8 cells<br><br>Ex/Em filter cube of Nikon Epifluorescence Microscope: Ex: 560/25 Em: 607/40	Ex: 570 Em: 580	Ex: 544 Em: 590/10
**MitoTracker orange CMTMRos**	Invitrogen (ThermoFisher) Ref: M7510	Thiol conjugation of cysteine residues	Membrane potential	DMSO	1 mM	-20 ˚C	100 nM	Incubation with 8x10E8 cells for 60 min at 37 ˚C, then 2x washed with growth medium by centrifugation (3 min, 3000 xg, RT)<br><br>Filter cubes of Nikon Epifluorescence Microscope: Ex: 560/25 Em: 607/40	Ex: 554 Em: 576	Ex: 544 Em: 590/10
**Rhodamine 123**	Invitrogen (ThermoFisher) Ref: R302	Always fluorescent, accumulates in cells due to its lipophilic and cationic properties	Membrane potential	DMSO	5.25 mM	-20 ˚C	15 µM	Incubation with 8x10E7 cells for 60 min at 37 ˚C, then 2x washed with growth medium by centrifugation (3 min, 3000 xg, RT) prior to concentration to 8x10E8 cells<br><br>Filter cubes of Nikon Epifluorescence Microscope: Ex: 485/20 Em: 521/30	Ex: 507 Em: 527	Ex: 485/12 Em: 520
**Syto9 (*Bac*Light LIVE/DEAD kit)**	Invitrogen (ThermoFisher) Ref: L7012	DNA intercalation	All cells (membrane permeable probe; commonly interpreted as live cells)	Ready to use solution (DMSO)	3.34 mM	-20 ˚C	7 µM	Incubation with 8x10E8 cells for 15 min at RT<br><br>Laser of Zeiss LSC Microscope: Ex: 488 Em: 490/30	Ex: 480 Em: 500	Ex: 485/12 Em: 520
**Propidium Iodide (*Bac*Light LIVE/DEAD kit)**	Invitrogen (ThermoFisher) Ref: L7012	DNA intercalation	Damaged/highly permeable cell membranes (membrane-impermeable probe; commonly interpreted as dead cells)	Ready to use solution (DMSO)	20 mM	-20 ˚C	40 µM	Incubation with 8x10E8 cells for 15 min at RT<br><br>Laser of Zeiss LSC Microscope: Ex: 561 Em: 561/758	Ex: 530 Em: 625	Ex: 530/10 Em: 620/10

*The resazurin reduction can also be tracked using colorimetric methods, measuring resazurin absorbance at 600 nm and resorufin at 570 nm.

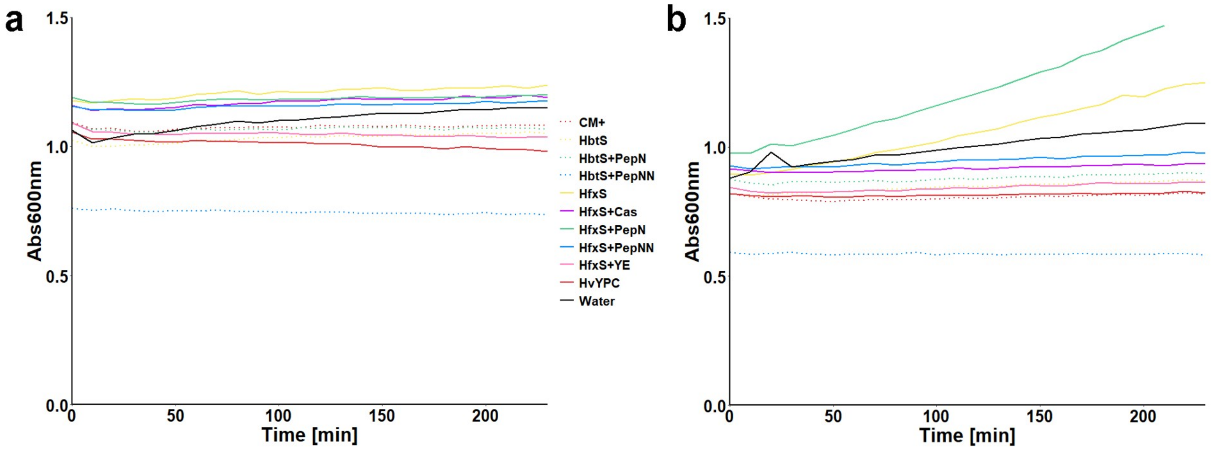

Figure S1: Effects of high-salt growth media composition on bulk measurements of alamarBlue. Cell-free solutions were prepared representing complex growth media (HvYPC for *Hfx. volcanii* and CM+ for *Hbt. salinarum*) as well as deconstructed media solutions: basal salt solutions without organics (HfxS and HbtS), basal salts with non-neutralized peptone (HfxS/HbtS+PepNN), and basal salts with neutralized peptone (HfxS/HbtS+PepN). For *Hfx. volcanii* only, basal salts with yeast extract (HfxS+YE) or with casamino acids (HfxS+Cas) (see Table S1 for compositions of each solution). Absorbance at 600 nm (Abs600nm) of each cell-free solution was measured over time to identify formation of resorufin (pink color, maximal absorption at 570nm) following the reduction of resazurin (blue color, maximal absorption at 600 nm). Freshly made solutions were first incubated with alamarBlue and the Abs600nm measured (panel a). A second set of solutions were stored for 3 mo at RT and incubated with alamarBlue (panel b) to account for potential degradative processes occurring during extended storage periods for growth media. All salt-based solutions with alamarBlue showed lower absorption values after 3 mo.

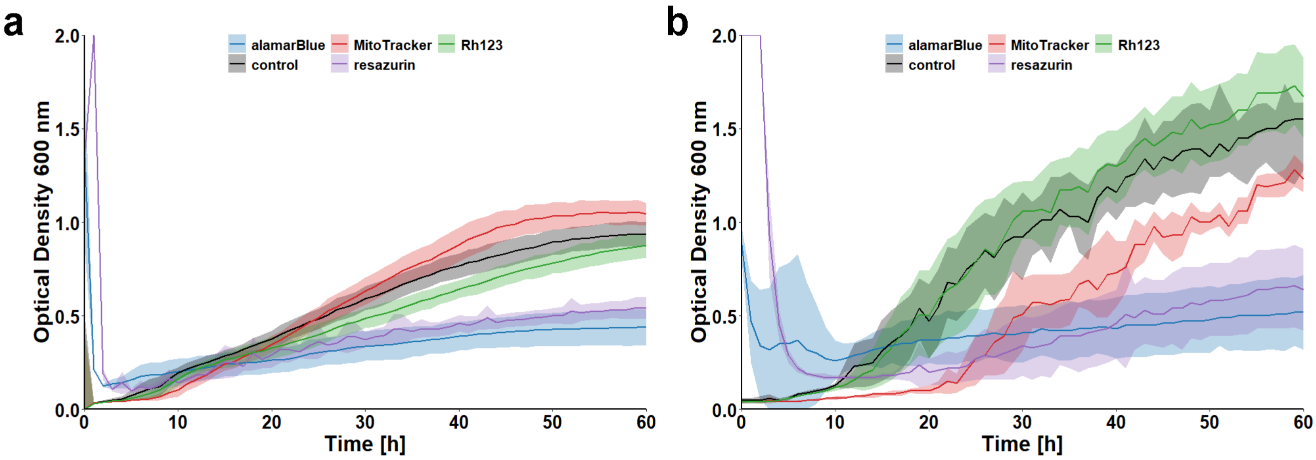

Figure S2: Growth impact of fluorescent probes on (a) *Hbt. salinarum* and (b) *Hfx. volcanii*. Organisms were grown in complex medium with alamarBlue, resazurin, MitoTracker Orange CMTMRos, Rhodamine 123 (Rh123) and without fluorophores (control) in 96-well plates to measure OD600nm over time with blank-corrected OD600nm. The initial increase in OD600nm in the presence of alamarBlue and resazurin for both species (time: 1 h to 4 h) was due to absorption overlap at 600 nm of cells and blue resazurin (prior to its reduction by redox-active cells to the pink resorufin).

Table S3: Results of the two-way ANOVA with Tukey post-hoc analysis on growth curves of *Hbt. salinarum* or *Hfx. volcanii* with different fluorescent probes (curve shown in Figure S2) in comparison to their respective control culture without any fluorescent probe.

Sample 1	Sample 2	p-value in post-Hoc analysis	Result
alamarBlue – Hfx	Control – Hfx	< 0.0001	Significantly different to control
alamarBlue – Hbt	Control – Hbt	< 0.0001	Significantly different to control
resazurin – Hfx	Control – Hfx	0.0018	Significantly different to control
resazurin – Hbt	Control – Hbt	< 0.0001	Significantly different to control
MitoTracker – Hfx	Control – Hfx	0.0006	Significantly different to control
MitoTracker – Hbt	Control – Hbt	0.5538	No significance
Rhodamine123 – Hfx	Control – Hfx	1.0	No significance
Rhodamine123 – Hbt	Control – Hbt	0.1336	No significance

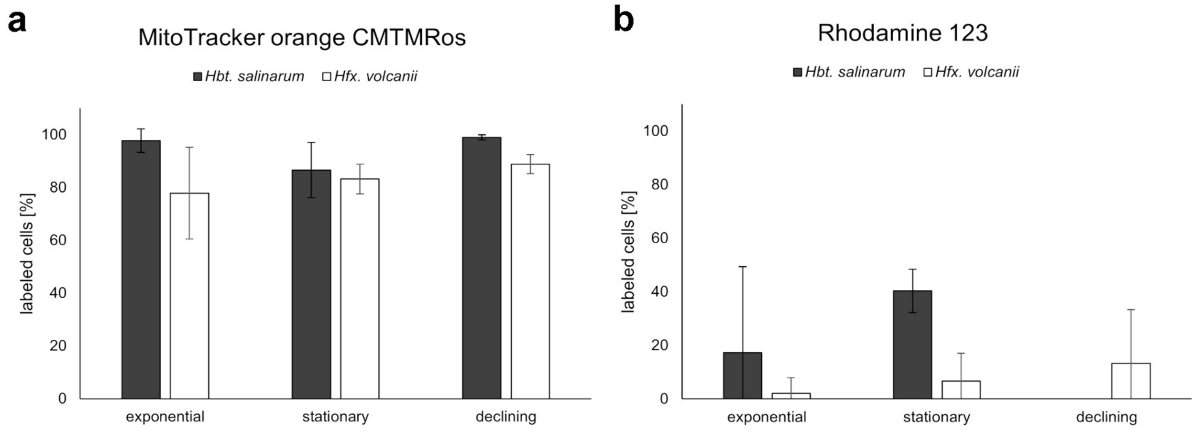

Figure S3: Percentage of *Hbt. salinarum* (grey) and *Hfx. volcanii* (white) labeled with either MitoTracker Orange CMTMRos or Rhodamine 123, as a function of growth phase. Cells were manually counted (biological triplicates with fields of view including >10 cells each) from epifluorescence images and percentage of labeled cells was calculated as the total number of cells in fluorophore image divided by the total number of cells in correlating brightfield image. Error-bars show standard deviation of samples. Differences in labeled cell numbers were compared between the growth phases for each organism with a one-way ANOVA and the Tukey post-hoc test. Only the decrease in % labeled cells with Rhodamine 123 in *Hbt. salinarum* from stationary to declining growth phase is significant ($p = 0.034$).

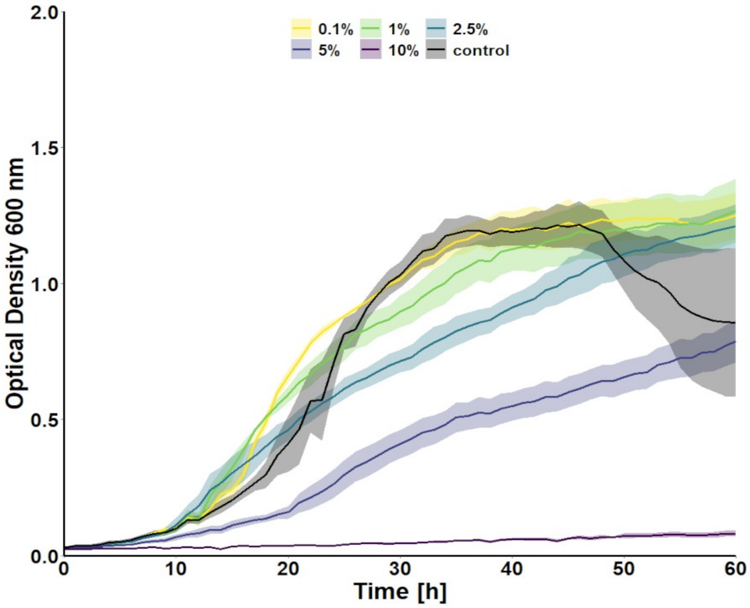

Figure S4: Growth impact of DMSO on *Hbt. salinarum* in complex medium. DMSO concentrations of 10 %, 5 %, 2.5 %, 1 %, 0.1 % [v/v] or without DMSO (control) were added to cultures of *Hbt. salinarum*. Growth was measured as OD600nm over shaking incubation. Growth inhibition could be observed for concentrations of 2.5 % DMSO and higher.

Table S4: Results of the two-way ANOVA with a Tukey post-hoc analysis on growth curves of *Hbt. salinarum* in DMSO (growth curves shown in Figure S4) in comparison to a *Hbt. salinarum* control culture that was incubated without any DMSO.

Sample tested against *Hbt. salinarum* control	P-value in post-Hoc analysis	Significance
10 % DMSO	< 0.0001	Significantly different to control
5 % DMSO	< 0.0001	Significantly different to control
2.5 % DMSO	0.9871	No significance
1 % DMSO	0.4316	No significance
0.1 % DMSO	0.0948	No significance

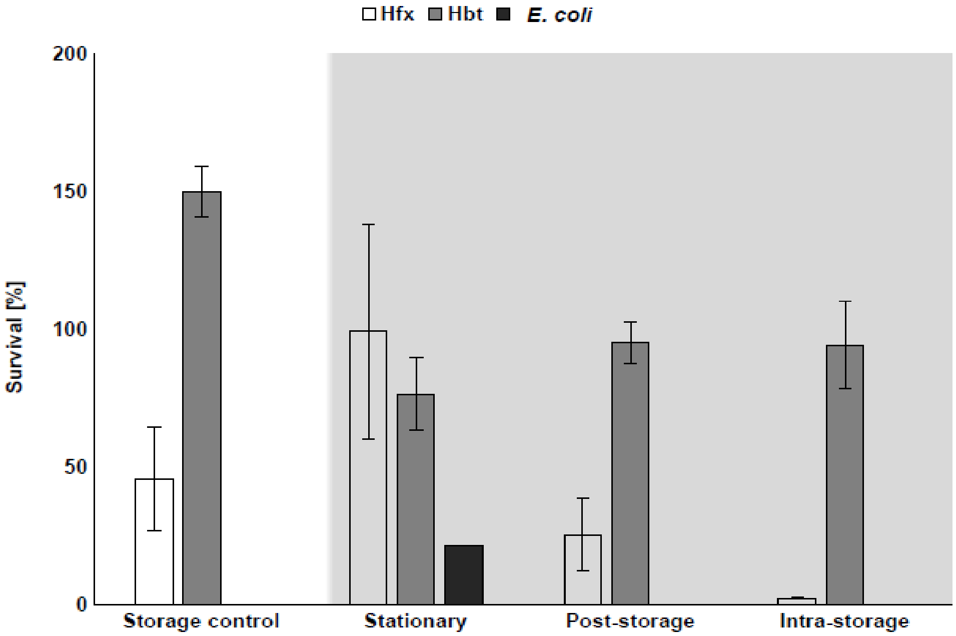

Figure S5: Comparison of colony forming units (CFU) for *Hbt. salinarum* (grey), *Hfx. volcanii* (clear) and *E. coli* (dark grey) with respect to both the physiological condition of the cells and the time of incubation with the LIVE/DEAD kit probes (SYTO 9, PI). Light-grey background indicating labeling experiments. Cells were either labeled immediately after reaching stationary growth phase by using a 15 min incubation (see table S2) with both probes ('stationary'), or cultures were stored for an additional 5 d reaching a decline phase to simulate entombment in halite crystals. These cells were either labeled after the secondary 5-day incubation ('post-storage') or continuously exposed to SYTO 9 and PI throughout this secondary 5-day incubation ('intra-storage') light-protected for the fluorescent probes. 'storage control' (clear background) presenting the control-incubation over 5 days. Following each treatment cells were plated on nutrient agar and incubated at 37 °C. Percentage survival was calculated as CFU for each incubation condition divided by CFU of unlabeled freshly stationary cultures. An increased number of CFU was observed for storage control samples of *Hbt. salinarum*, indicating cell proliferation, albeit slow, over the 5 d storage. Sample treatment scheme is shown in Figure S5.1.

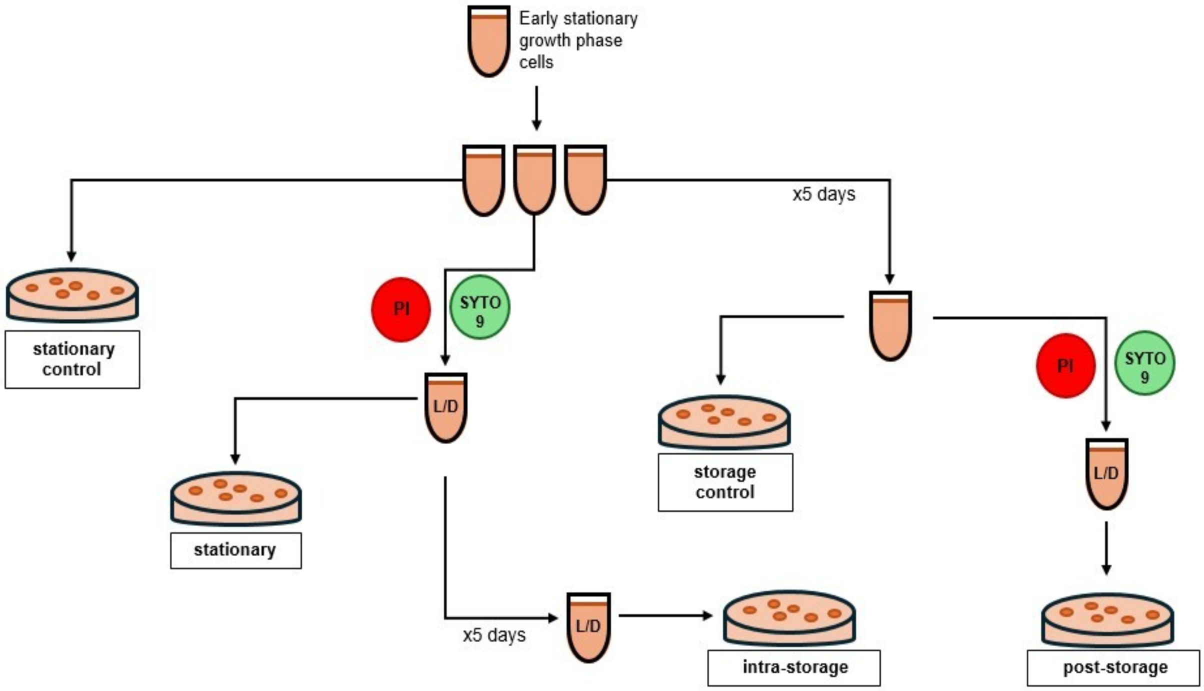

Figure S5.1: Method flow-chart of LIVE/DEAD kit testing with haloarchaeal cells. Early stationary growth phase cells in liquid cultures were split into three aliquots per sample. The first replicate (stationary control) was immediately plated (as serial dilution) on complex medium agar (CMA) and incubated at 37 °C to manually count colony forming units (CFU). The second replicate was labeled for 15 min at RT with Propidium Iodide (PI) and SYTO 9, the fluorescent probe components of the LIVE/DEAD kit, then either plated on CMA (stationary) or incubated over 5 days at RT under light-protection and then plated on CMA (intra-storage). The third replicate was incubated over 5 days (RT, light protected) and then either plated on CMA (storage control) or labeled for 15 min at RT with PI and SYTO 9 and then plated on CMA (post-storage).
